# Predictors of Lung Adenocarcinoma With Leptomeningeal Metastases: A 2022 Targeted-Therapy-Assisted molGPA Model

**DOI:** 10.3389/fonc.2022.903851

**Published:** 2022-06-10

**Authors:** Milan Zhang, Jiayi Tong, Weifeng Ma, Chongliang Luo, Huiqin Liu, Yushu Jiang, Lingzhi Qin, Xiaojuan Wang, Lipin Yuan, Jiewen Zhang, Fuhua Peng, Yong Chen, Wei Li, Ying Jiang

**Affiliations:** ^1^ Department of Neurology, Henan Joint International Research Laboratory of Accurate Diagnosis, Treatment, Research and Development, Henan Provincial People’s Hospital, People’s Hospital of Zhengzhou University, Zhengzhou, China; ^2^ Department of Biostatistics, Epidemiology and Informatics, University of Pennsylvania, Philadelphia, PA, United States; ^3^ Division of Public Health Sciences, Washington University School of Medicine in St. Louis, St Louis, MO, United States; ^4^ Department of Neurology, The Third Affiliated Hospital of Sun Yat-sen University, Guangzhou, China

**Keywords:** leptomeningeal metastases, lung adenocarcinoma, molGPA model, overall survival, targeted therapy

## Abstract

**Objective:**

To explore prognostic indicators of lung adenocarcinoma with leptomeningeal metastases (LM) and provide an updated graded prognostic assessment model integrated with molecular alterations (molGPA).

**Methods:**

A cohort of 162 patients was enrolled from 202 patients with lung adenocarcinoma and LM. By randomly splitting data into the training (80%) and validation (20%) sets, the Cox regression and random survival forest methods were used on the training set to identify statistically significant variables and construct a prognostic model. The C-index of the model was calculated and compared with that of previous molGPA models.

**Results:**

The Cox regression and random forest models both identified four variables, which included KPS, LANO neurological assessment, TKI therapy line, and controlled primary tumor, as statistically significant predictors. A novel targeted-therapy-assisted molGPA model (2022) using the above four prognostic factors was developed to predict LM of lung adenocarcinoma. The C-indices of this prognostic model in the training and validation sets were higher than those of the lung-molGPA (2017) and molGPA (2019) models.

**Conclusions:**

The 2022 molGPA model, a substantial update of previous molGPA models with better prediction performance, may be useful in clinical decision making and stratification of future clinical trials.

## Introduction

Leptomeningeal metastases (LM) refers to the seeding of tumor cells within the subarachnoid space and leptomeninges. It occurs in up to 10% of adult patients with solid tumors, especially melanoma, breast cancer, and non-small cell lung cancer (NSCLC) (1, 2). The incidence of LM as a devastating complication of NSCLC is increasing, especially in patients with targeted molecule-driven mutations (3, 4). Lung adenocarcinoma, which is the main component of NSCLC, is more likely to develop LM. Molecular targeted therapy has shown antitumor activity in central nervous system metastases, with median overall survival ranging from 1 to 3 months for historical treatments and 3 to 11 months for new treatments (4, 5). Therefore, patients with lung adenocarcinoma have a greater risk of developing sequelae of advanced diseases in the future, such as brain metastasis (BM) and LM. These trends, coupled with the wide application of magnetic resonance imaging (MRI), indicate that an increasing number of patients will be diagnosed with LM in the next few years.

Some existing studies have focused on predicting the occurrence of heterogeneous BM. The Radiation Therapy Oncology Group (RTOG) database was used to generate the recursive partitioning analysis (RPA) classes which were modified in 2012 (modified RPA) (6–8). RPA is a prognostic index that is divided into three classes based on age, Karnofsky performance status (KPS), control of primary tumor, and extracranial metastases (ECM). The graded prognostic assessment (GPA) index was developed in 2007 and revised in 2017 to form a lung-molGPA model using age, KPS, ECM, number of BM, and gene status to define four disease classes, with median survival ranging from 3.0 to 14.8 months (9–12). In 2019, another molGPA model was developed to predict LM using factors, such as KPS, ECM, and gene status (13).

In both the lung-molGPA (2017) and molGPA (2019) models, gene mutation status was identified as a significant prognostic factor (11, 12). From a clinical perspective, gene mutation status, which indicates molecular-targeted therapy, also has a significant impact on the treatment of EM and LM. However, the efficacy of third-generation targeted drugs has led to revolutionary development compared to first- or second-generation targeted therapeutic approaches (2–5, 14, 15). According to the BLOOM and AURA studies (5, 14, 15), the third-generation epidermal growth factor receptor (*EGFR*)*-*tyrosine kinase inhibitor (TKI) resulted in a significantly improved median overall survival (OS) of 11.0-18.8 months compared to even higher doses of first- or second-generation *EGFR* TKIs with a median OS of 3.1-6.2 months (2). The differences in efficacy between generations of targeted therapy may affect the prediction efficiency of the molGPA models. Therefore, in this study, we compared the effects of gene mutation status and targeted therapy on survival, and developed a novel 2022 lung-molGPA for the patients of lung adenocarcinoma with LM.

To the best of our knowledge, no studies have been conducted to predict the survival of lung adenocarcinoma with LM using targeted therapy; moreover, the use of machine learning methods, such as random forests, is lacking. Therefore, this study aimed to fill this research gap and study the role of targeted therapy in the prediction of lung adenocarcinoma with LM using both conventional molGPA and random forest models.

## Methods

### Study Design and Samples

The study was conducted in accordance with the principles of the Declaration of Helsinki, and the protocol was approved by the Medical Ethics Committee of Henan Provincial People’s Hospital (approval number: 2017-28). All study participants provided written informed consent for the research and publication.

**Figure 1:**
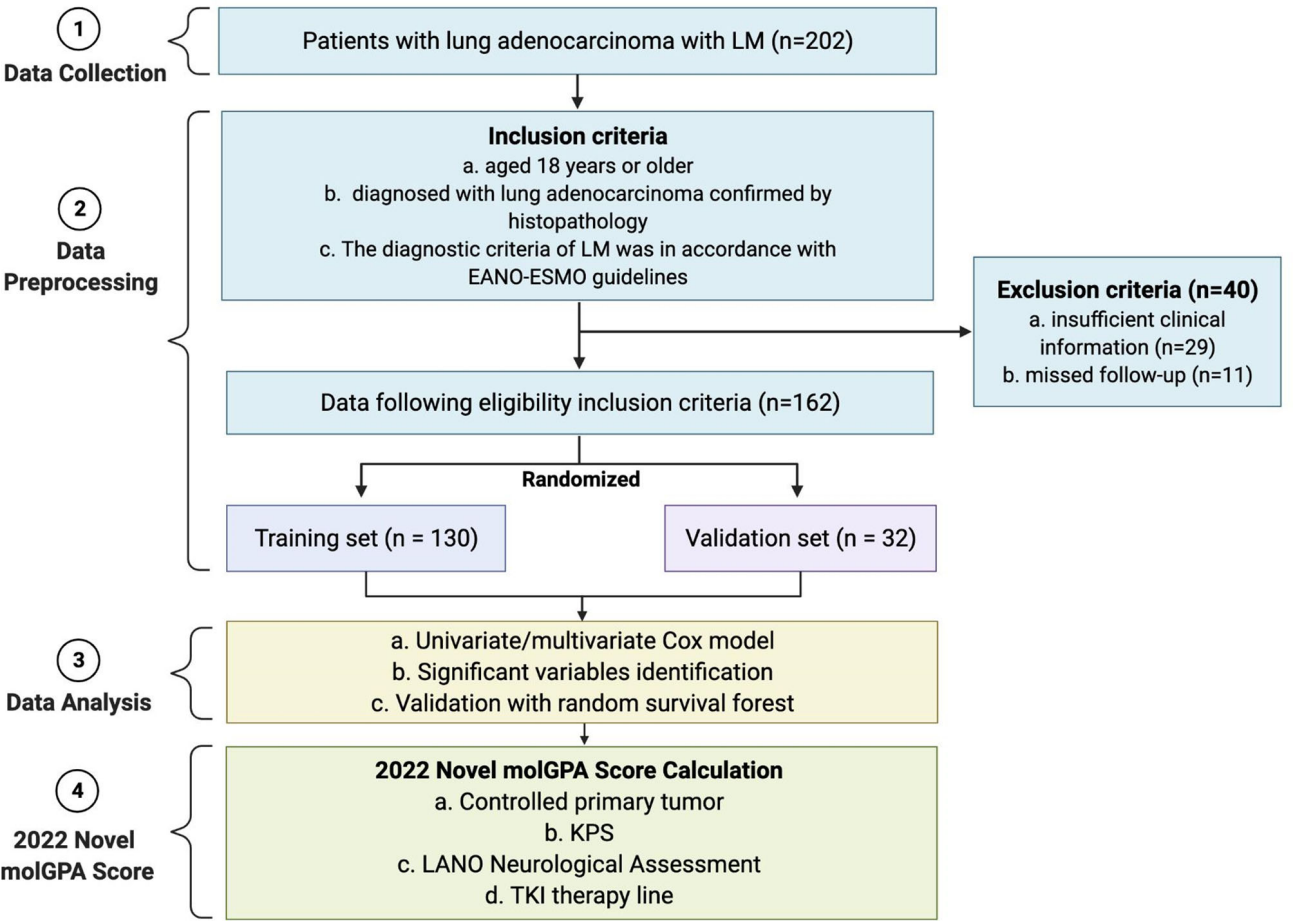
Flow diagram of the enrollment of patients with lung adenocarcinoma with LM, and pipeline of data analysis to get the 2022 molGPA score. LM, leptomeningeal metastases; EANO-ESMO, European Association of Neuro-Oncology-European Society for Medical Oncology. KPS, Karnofsky performance status; LANO, Leptomeningeal Assessment in Neuro-Oncology; TKI, tyrosine kinase inhibitor; GPA, Graded Prognostic Assessment.

We collected data from 202 lung adenocarcinoma patients with LM, enrolled between April 2017 and January 2022, at Henan Provincial People’s Hospital, Zhengzhou, China. The inclusion criteria were as follows: (i) ≥ 18 years; (ii) diagnosis of lung adenocarcinoma confirmed by histopathology; and (iii) LM diagnosis ascertained according to the NCCN guidelines and the European Association of Neuro-Oncology-European Society for Medical Oncology (EANO-ESMO) guidelines (16). According to the Leptomeningeal Assessment in Neuro-Oncology (LANO) neurological assessment in LM (**Supplement Table 1**) (17), all patients underwent complete work up, including standardized neurological examination, brain and spine MRI, CSF analysis, during hospitalization. Patients with insufficient clinical information (n=29) or missing follow-up data (n=11) were excluded. Finally, 162 patients were included in the study cohort and randomly assigned to the training (80%, n = 130) and validation (20%, n = 32) sets (**Figure 1**).

Baseline clinicopathological characteristics of each patient were obtained from their medical records; they included age, sex, smoking status, ECM, controlled primary tumor, clinical presentations, KPS, gene profiles of *EGFR* mutation and *ALK* alteration, ThinPrep cytologic test (TCT), and brain and spine MRI. Treatments including TKI therapy, chemotherapy, bevacizumab, surgery, radiotherapy, intrathecal chemotherapy, and immune checkpoint inhibitors were included in the study. Controlled primary tumor was defined as remission or stable disease, without any clinical, radiologic, or laboratory findings suggestive of tumor progression at 2 months (6, 7, 18). The overall survival (OS) was defined as the time from diagnosis of LM to death.

#### Statistical Analysis

Missing values were imputed for variables with small missing proportion. Continuous variables, that is, CSF white blood cells, protein, and glucose, were transformed by taking the logarithm. Other continuous variables were categorized based on clinical reasoning and statistical methods. KPS status was divided into 3 groups: < 60 (high-risk group), 60-70 (moderate-risk group), and 80-100 (low-risk group). Age was dichotomized using a 65-year cutoff. Univariate Cox models were performed on the training set (n = 130), covering baseline characteristics, clinical symptoms, brain and spinal MRI, CSF analysis and treatment, to identify statistically significant variables. With significant variables in the univariate analysis, a multivariate Cox model was fitted to the training set to select significant predictors to construct the prognostic model.

We further utilized the random survival forest method to validate the selected predictors using the Cox model. In addition to the clinical prediction because of the high variance bias trade-of capability, Random survival forests (19, 20) method is also usually used to select the most important variables that are linked with the time-to-event outcome (i.e., OS). Given these advantages of random survival forests, we first utilized all variables in the model to identify those with positive importance values. With the top variables, we performed the random survival forest method again to select significant variables, and compared them with those from the Cox model. Furthermore, the C-index of the prognostic model constructed using the top variables was calculated.

We constructed a novel molGPA model (2022) using statistically significant variables. The model was then used to predict the OS of LM with lung adenocarcinoma cancer. The C-index of the prognostic model was calculated and compared with the lung-molGPA (2017, **Supplemental Table 2**) and molGPA (2019) models (**Supplemental Table 3**) by taking the average of the C-index values from the randomly split training and validation sets 100 times. Missing values were imputed for variables with small missing proportion using R package *mice* with default settings (e.g., the number of multiple imputations is 5) (21). All analyses were conducted in R software using the *mice* package (21) for multiple imputation, *survival* package (22) for Cox model and C-index, and the *randomForestSRC* package (19, 20) for random forest. The R code for analysis is available on the Github Page: https://github.com/Penncil/A-2022-Targeted-therapy-assisted-molGPA-.

## Results

### Clinicopathological Characteristics of the Patients

The baseline clinical characteristics of patients in the training and validation cohorts are presented in **Table 1**. There were no significant differences in sex, age, smoking status, clinical symptoms, KPS, gene mutation status, LANO neurological assessment, ECM, BM, controlled primary tumor, TCT, and brain or spinal MRI between the training and validation sets. The median time from NSCLC to LM diagnosis was 10 (range: 0-120) months and 6 (range: 0-100) months in the two cohorts, respectively. Missing values of gene mutation status (11.1% missing), lumbar puncture pressure (29.6% missing), CSF white blood cells (29.6% missing), protein (29.6% missing), and glucose (29.6% missing) were imputed.

**Table 1:** Demographic and clinical characteristics of the 162 lung adenocarcinoma patients with LM.

Characteristic	Patients, No. (%)		*p*-value
	Training set (n = 130)	Validation Set (n = 32)	
**Age**			0.30
≤65	90 (69.2)	25 (78.1)	
>65	40 (30.8)	7 (21.9)	
**Sex**			0.54
Male	57 (43.8)	16 (50.0)	
Female	73 (56.2)	16 (50.0)	
**Smoke**			0.89
No	95 (73.1)	23 (71.9)	
Yes	35 (26.9)	9 (28.1)	
**Median time diagnosis to LM (median, range)**	10 (0, 120)	6 (0, 100)	0.52
**Clinical symptoms**			
Headache	97 (74.6)	21 (65.7)	0.34
Abnormal levels of consciousness and behavior	35 (26.9)	7 (21.9)	0.55
Cognitive impairment	25 (19.2)	4 (12.5)	0.33
Epilepsy	26 (20.0)	9 (28.1)	0.36
Cranial neuropathies	41 (31.5)	12 (37.5)	0.54
Spinal neuropathies	13 (10.0)	2 (6.3)	0.46
**KPS at diagnosis of LM**			0.11
<60	50 (38.5)	7 (21.9)	
60-70	42 (32.3)	13 (40.6)	
80-100	38 (29.2)	12 (37.5)	
Gene status*			0.11
*EGFR*/*ALK* mutation	103 (79.2)	25 (78.1)	
Wild type	13 (10.0)	6 (18.8)	
Unknown	14 (10.8)	1 (3.1)	
**LANO neurological assessment**			0.44
≥6	34 (26.2)	7 (21.9)	
3-5	22 (16.9)	5 (15.6)	
≤2	74 (56.9)	20 (62.5)	
**Extracranial metastases**			0.52
No	16 (12.3)	4 (12.5)	
Yes	114 (87.7)	28 (87.5)	
**Brain metastasis**			0.65
No	51 (39.2)	14 (43.8)	
Yes	79 (60.8)	18 (56.5)	
**Controlled primary tumor**			0.33
No	82 (63.1)	23 (71.9)	
Yes	48 (36.9)	9 (28.1)	
Thinprep cytologic test*			0.54
Positive	99 (76.2)	18 (56.2)	
Negative/Unknown	31 (23.8)	14 (43.8)	
Brain and spinal MRI*			0.46
Positive	117 (90.0)	30 (93.8)	
Negative	13 (10.0)	2 (6.2)	
**TKI therapy line**			0.15
≤2nd	45 (34.6)	13 (40.6)	
3rd	58 (44.6)	12 (37.5)	
**Treatments before LM**			
TKIs	77 (59.3)	15 (46.9)	0.22
Chemotherapy	60 (46.2)	14 (43.8)	0.81
Bevacizumab	13 (10.0)	3 (9.4)	0.92
Without treatments	37 (28.5)	16 (50.5)	0.54
**Treatments for LM**			
TKIs	103 (79.2)	24 (75.0)	0.40
Chemotherapy	66 (50.7)	19 (59.4)	0.39
Bevacizumab	38 (29.2)	7 (21.9)	0.12
Operation	20 (15.4)	8 (25.0)	0.26
Radiotherapy	24 (18.5)	4 (12.5)	0.39
Intrathecal chemotherapy	22 (16.9)	3 (9.4)	0.23
Immunotherapy	5 (3.8)	2 (6.3)	0.61

LM, leptomeningeal metastases; KPS, Karnofsky performance status; EGFR, epidermal growth factor receptor; ALK, anaplastic lymphoma kinase; TKI, tyrosine kinase inhibitor; LANO, Leptomeningeal Assessment in Neuro-Oncology; ECM, extracranial metastases; BM, brain metastasis; MRI, magnetic resonance imaging. *Missing values: gene mutation status (11.1% missing), thinprep cytologic test (29.6% missing), brain and spinal MRI (2.4 % missing).

### Treatment

As shown in **Table 1**, prior to LM diagnosis, 77/130 and 15/32 patients had undergone TKI therapy, 60/130 and 14/32 patients received cytotoxic chemotherapy, and 37/132 and 16/32 patients initially diagnosed with LM did not receive any treatment in the two cohorts, respectively.

*EGFR*/*ALK* alterations were detected in of 103/132 and of 25/32 patients in the two cohorts, respectively. Among those who received *EGFR*-TKI or *ALK*-TKI therapy after LM diagnosis, some patients (45/103 and 13/25) received first- or second-generation TKIs (gefitinib, erlotinib, icotinib, afatinib, crizotinib, alectinib, and ceritinib), while other patients (58/103 and 12/25) received third-generation TKIs (osimertinib and lorlatinib).

### Survival Analysis *via* Cox Regression Model

As shown in **Table 2**, the univariate Cox proportional hazard regression models showed that age, KPS, controlled primary tumor, gene mutation status, CSF chloride, LANO neurological assessment, and TKI therapy line were significantly associated with OS (all with p < 0.05). There was no significant correlation between ECM, BM, MRI, and CSF white blood cells, protein levels, glucose levels and OS (p > 0.05). With the significant variables identified by the univariate Cox model, we further fitted the multivariate Cox model and found that KPS (HR = 0.47, 95% CI [0.22, 1.00], p=0.046), LANO neurological assessment (HR = 1.12, 95% CI [1.06, 1.17], p < 0.001), and TKI therapy (HR = 0.24, 95% CI [0.08, 0.71], p = 0.01) were significantly associated with OS in patients with LM. Controlled primary tumors may be a significant factor for OS (HR = 0.66, 95% CI [0.40, 1.06], p = 0.09), with a p-value at the boundary. However, gene mutation status was not statistically significant in the multivariate Cox model (p = 0.26). Considering the correlation between gene mutation status and TKI therapy line (3, 4), we fitted the multivariate Cox model again by including the gene mutation status only (**Supplemental Table 4**). The results showed that the p-value of the gene mutation status was 0.07.

**Table 2:** Univariate and multivariate Cox regression analysis of overall survival of the training set.

Variables	Model			
	Univariate analysis		Multivariate analysis	
	HR (95% CI)	*p* value	HR (95% CI)	*p* value
**Age, year**				
>65	1 [Reference]		1 [Reference]	
≤65	0.63 (0.41, 0.96)	0.03	0.96 (0.60, 1.53)	0.88
**Sex**				
Male	1 [Reference]			
Female	0.88 (0.59, 1.32)	0.53		
**KPS**				
<60 (reference level)	1 [Reference]		1 [Reference]	
60-70	0.39 (0.25, 0.63)	<0.01		
80-100	0.21 (0.12, 0.36)	<0.01	0.47 (0.22, 1.00)	<0.05
**Concurrent BM**				
No	1 [Reference]			
Yes	0.92 (0.61, 1.38)	0.67		
**Number of BM**				
	0.97 (0.88, 1.06)	0.46		
**Concurrent ECM**				
No	1 [Reference]			
Yes	1.26 (0.70, 2.26)	0.44		
**Controlled primary tumor**				
No	1 [Reference]		1 [Reference]	
Yes	0.55 (0.36, 0.84)	0.01	0.66 (0.40, 1.06)	0.09
**Mutation status**				
No mutation	1 [Reference]		1 [Reference]	
*EGFR*/*ALK* mutation	0.45 (0.27, 0.77)	<0.01	2.05 (0.73, 5.77)	0.26
**LANO neurological assessment**				
	1.13 (1.10, 1.17)	<0.01	1.12 (1.06, 1.17)	<0.01
**CSF analysis**				
Chloride	0.97 (0.95, 1.00)	0.05		
Thinprep cytologic test	1.00 (0.99, 1.01)	0.08		
**Brain and spinal MRI**				
Negative	1 [Reference]			
Positive	1.04 (0.54, 2.01)	0.91		
**TKI therapy line**				
No therapy	1 [Reference]			
1^st^ or 2^nd^	0.52 (0.30, 0.90)	<0.01		
3^rd^	0.31 (0.18, 0.54)	<0.01	0.24 (0.08, 0.71)	0.01

LM, leptomeningeal metastases; KPS, Karnofsky performance status; EGFR, epidermal growth factor receptor; ALK, anaplastic lymphoma kinase; TKI, tyrosine kinase inhibitor; LANO, Leptomeningeal Assessment in Neuro-Oncology; ECM, extracranial metastases; BM, brain metastasis; MRI, magnetic resonance imaging.

### Random Survival Forest Model

**Figure 2:**
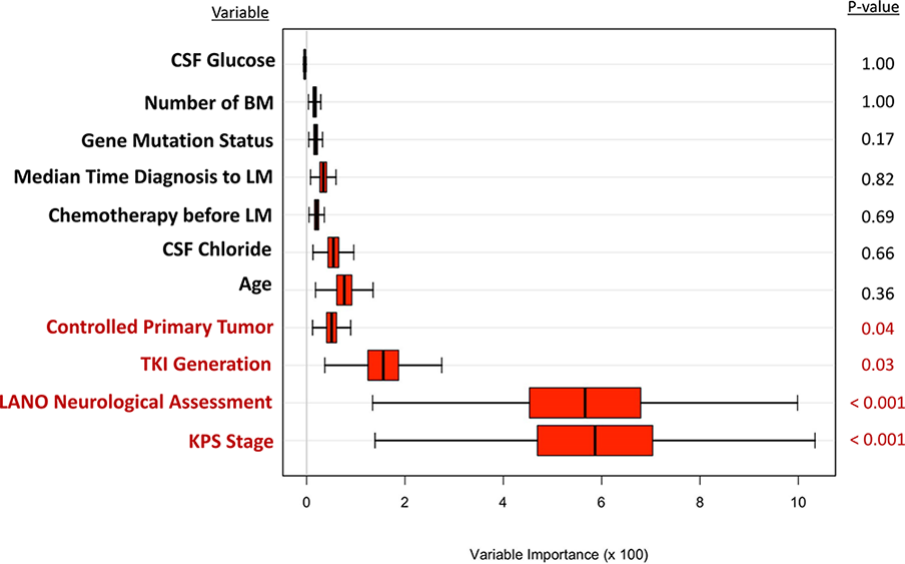
The random forest model for predicting survival of lung adenocarcinoma with LM. LM, leptomeningeal metastases; KPS, Karnofsky performance status; LANO, Leptomeningeal Assessment in Neuro-Oncology; TKI, tyrosine kinase inhibitor; CSF, cerebrospinal fluid; GPA, Graded Prognostic Assessment.

A random survival forest model for predicting survival of patients with lung adenocarcinoma with LM was fitted to validate the results of the Cox model. As shown in **Figure 2**, candidate predictor variables were ranked according to their importance in terms of prognostic accuracy. Among these variables, the top four variables, which included KPS, LANO neurological assessment, TKI therapy line, and controlled primary tumor with p-values less than 0.05, were consistent with those identified by the multivariate Cox proportional hazard regression model.

### Establishment and Internal Validation of the 2022 molGPA Model

**Figure 3:**
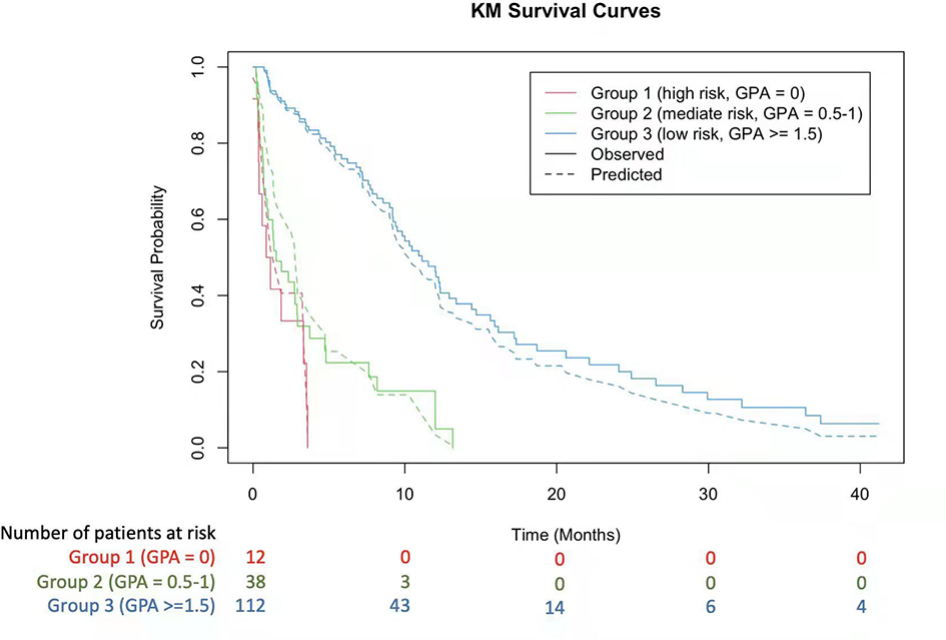
Kaplan-Meier Curves Showing Survival using the 2022 molGPA for lung adenocarcinoma with LM. GPA, Graded Prognostic Assessment.

By selecting statistically significant variables with the multivariate Cox and random forest models, we developed a novel molGPA model (2022) for LM of lung adenocarcinoma cancer using four parameters: controlled primary tumor, KPS, LANO neurological assessment, and TKI therapy line (**Table 3**). Factors with larger effect sizes were given a maximum score of 1.0, including KPS from 80 to 100 (HR, 0.47 vs KPS < 60), LANO neurological assessment ≤2 (HR, 1.12) and 3^rd^-TKI therapy line (HR, 0.42 vs no TKI therapy), with higher scores corresponding to better prognosis. The controlled primary tumor had a smaller effect size (HR, 0.66), with a maximum score of 0.5. The model had a maximum score of 3.5; the higher the score, the lower the risk was. The targeted-therapy-assisted molGPA score was calculated for each patient and categorized into three groups: molGPA 0 (group 1, high risk), 0.5-1.0 (group 2, mediate risk), and ≥ 1.5 (group 3, low risk). For all the patients, the median OS for the three subgroups was 1.01 (95% CI [0.09, 3.58]), 1.45 (95% CI [0.24, 12.09]), and 8.02 (95% CI [0.98, 38.13]) months, respectively. The Kaplan-Meier curve for predicting the OS probability of the study population is shown in **Figure 3**, which demonstrates significant separation among the three groups.

**Table 3:** The scoring criteria of the 2022 novel molGPA.

Prognostic Factor	2022 Novel molGPA Scoring Criteria		
	0	0.5	1
Controlled primary tumor	No	Yes	NA
KPS	<60	60-70	80-100
LANO neurological assessment	≥6	3-5	≤2
TKI therapy line	No	1^st^ and 2^nd^	3^rd^

GPA, graded prognostic assessment; KPS, Karnofsky performance status; LANO, Leptomeningeal Assessment in Neuro-Oncology; TKI, tyrosine kinase inhibitor.

### Model Evaluation

The previously reported lung-molGPA model (2017) (12) and molGPA model for LM (2019) (13) were tested in all patients. The C-index was calculated among the three models by taking the average of the C-index values from 100 randomly split training and validation sets. For each split, molGPA scores and concordance values were calculated. The higher the C-index, the better the survival time predicted by the model. The concordance results are shown in **Table 4**, where the average C-index of this model on the training set was 0.710 (95% CI [0.69, 0.73]), which is 7.00% higher than that of the lung-molGPA (2017) and 5.5% higher than that of molGPA (2019) models. The C-index of the model on the validation set was 0.714 (95% CI [0.63, 0.80]), which was 8.3% higher than that of the lung-molGPA (2017) and 5.9% higher than that of the molGPA (2019) models.

**Table 4:** Concordance results of three GPA models.

Models	Training set (95% CI)	Validation Set (95% CI)
Lung-molGPA (2017)	0.66 (0.64, 0.69)	0.66 (0.56, 0.76)
MolGPA for LM (2019)	0.67 (0.65, 0.70)	0.67 (0.58, 0.77)
Novel molGPA (2022)	0.71 (0.69, 0.73)	0.71 (0.63,0.80)

LM, leptomeningeal metastases; GPA, graded prognostic assessment.

We also calculated the C-indices of the random survival-forest-derived prognostic model. The C-index for the training set (80% of the cohort) was 0.722 (95% CI [0.69, 0.74]), and 0.714 (95% CI [0.60, 0.84]) for the validation set (20% of the cohort). The C-index of the training set was slightly larger (1.7%) than that of the Cox-based prognostic model. This is because the prognostic model with the random survival forest method included all variables listed in **Figure 2** rather than only the top four variables. The C-indices of the validation set of these two prognostic models (i.e., Cox-based and random-survival-forest-based) were the same (i.e., C-index = 0.714).

## Discussion

To the best of our knowledge, this is the first attempt to construct a 2022 targeted-therapy-assisted molGPA for LM of lung adenocarcinoma using a multivariate Cox proportional hazard regression model and the random survival forest method. The molGPA model considered the following four variables: controlled primary tumor, KPS, LANO neurological assessment, and TKI therapy line. According to the molGPA model scores, patients were divided into three groups: 0 for high-risk, 0.5-1.0 for immediate high-risk, and ≥ 1.5 for low-risk. In both the training and validation sets, patients with an LM molGPA score ≥ 1.5 (low risk) were more likely to have a better OS than the other two groups. The C-index values of the proposed prognostic model for the training and validation sets were higher than those of the lung-molGPA (2017) and molGPA (2019) models (12, 13).

Our 2022 target-therapy-assisted molGPA for LM has several advantages. First, TKI therapy was used instead of gene mutations. The recent revolution in the treatment of patients with prognostic biomarkers has resulted in significant improvements in survival outcomes. As earlier mentioned, molecular markers were included as important factors in the lung-molGPA (2017) and molGPA (2019) models, and had been validated by several studies for its prognostic value in real-world cohorts (12, 13, 23, 24). However, in this study, gene mutation status was not statistically significant in the multivariate Cox model. Considering the correlation between gene mutation status and TKI therapy line, we fitted the multivariate Cox model again by including the gene mutation status only (**Supplemental Table 4**). The results showed a boundary p-value = 0.07 for the gene mutation status was 0.07, which suggested the possible prognostic value of mutated status in real-life cohorts. We further found that the TKI therapy line was a significant positive prognostic factor for LM, identified by the multivariate Cox and random forest models. The efficacy of first-generation *EGFR*-TKIs for *EGFR+* NSCLC remains poor because of low CSF penetration (25, 26). Although second-generation *EGFR*-TKIs, such as afatinib, can partially penetrate the blood-brain barrier, they exhibit no obvious advantages as treatment for LM (27). Osimertinib, an irreversible third-generation *EGFR* TKI, is highly effective in both untreated and previously treated patients with *EGFR*-mutant NSCLC, according to several encouraging international clinical trials (13–15, 28). For *ALK*+ NSCLC, lorlatinib is a novel, highly potent, brain-penetrant, third-generation *ALK* TKI with broad-spectrum potency against most known resistance mutations that can develop during treatment with existing first- and second-generation *ALK* TKIs; its efficacy is significant in BM and LM (29). Guttmann DM (30) also proposed that lung-molGPA is the critical first step in accurately defining the prognosis of patients with gene mutations; however, it also highlights the need for a prognostic index incorporating the utilization and timing of targeted therapy. Therefore, we considered that the TKI therapy line could be used as a significant positive prognostic factor in the prediction of LM.

The second advantage of our proposed molGPA is the use of the LANO assessment, a significant factor commonly used in clinical practice, which has never been considered by other prediction models. The LANO scorecard was formed by the Response Assessment in Neuro-Oncology (RANO) Leptomeningeal Metastasis Working Group, an international multidisciplinary group with the goal of improving response criteria and defining endpoints for neuro-oncology trials (17, 31). Although the LANO neurological assessment in LM has not yet been validated, the LANO scorecard generated a proposal for the response assessment in LM and has been widely used in international randomized clinical trials, including the BLOOM and AURA studies (5, 14, 15, 31, 32). Patients with LM from lung adenocarcinoma are treated in different departments, including neurology, oncology, and respiratory medicine. The LANO assessment (**Supplemental Table 1**) is a standardized assessment for neurological examination in the prediction model and is easily utilized by neurologists, oncologists, nurses, and physician assistants.

Third, KPS and controlled primary tumors, two clinically important significant prognostic factors, were considered in our molGPA model. Patients with a KPS score of 80-100 had better OS than those with KPS of 60-70 and KPS < 60. KPS was significantly associated with survival and was included in all the prediction models for BM and LM (6–10, 12, 13). A controlled primary tumor, requiring the estimation of control of systemic disease, was included in the RPA and basic score for BM (BSBM) models (6, 7, 18). In the study, controlled primary tumor had a p-value of 0.09 in the multivariate Cox model while a boundary p-value between 0.05 and 0.1 indicates weak evidence or a trend (33, 34). On the other hand, it was confirmed that in the full set data using random forest model, controlled primary tumor is significant with p=0.04. Because of the above two reasons, we considered controlled primary tumor as a significant factor and incorporated it into the proposed 2022 molGPA model. The controlled primary tumor was assigned a maximum of 0.5, based on its HR and statistical significance in the molGPA model for LM. Extracranial metastases were included in the Lung-molGPA (2017) and molGPA (2019) models (12, 13). However, in this study, extracranial metastases showed no statistical significance in Cox proportional hazard regression model and random forest analysis, which may be related to sample bias, requiring further analysis and verification of a larger sample of patients.

Our study had several limitations. First, it was a retrospective study from a single center and single ethnic population, which led to incompleteness of some variables. For example, forty-eight patients did not undergo lumbar puncture and had no available information on variables such as protein and white blood cells. However, the sensitivity analysis showed that excluding variables with missing data did not change our conclusions. Second, third-generation TKIs contain different *EGFR*- and *ALK-related* drugs, which may affect the prognostic effect of the TKI therapy line. Third, this study evaluated only lung cancer, not other solid cancers, such as melanoma and breast cancer, which are also common in LM. We intend to validate the 2022 molGPA model for LM with lung cancer and extend the model to other solid tumors in the further study.

## Conclusions

We developed a novel targeted-therapy-assisted 2022 molGPA model for predicting LM in lung adenocarcinoma by incorporating a TKI therapy line in addition to a controlled primary tumor, KPS, and LANO neurological assessment. The 2022 molGPA model has a better prediction performance and is a substantial update of previous molGPA models (11, 12). The 2022 molGPA model provides a user-friendly tool for estimating survival of lung adenocarcinoma patients with LM and may be useful in clinical decision-making and stratification of future clinical trials.

## Data Availability Statement

The datasets presented in this study can be found in online repositories. The names of the repository/repositories and accession number(s) can be found below: The git repository of this study pertinent code is at https://github.com/Penncil/2022_molGPA.

## Ethics Statement

The studies involving human participants were reviewed and approved by The medical ethics committee at the Henan Provincial People’s Hospital (ethics number: 2017-28). The patients/participants provided their written informed consent to participate in this study.

## Author Contributions

WL, YJ, JZ, FP and YC contributed to the conception and design of this study. WM, MZ, HL, YJ, LQ, XW and LY collected and organized the data. JT, CL, YC and MZ analyzed the data. MZ, JT, YJ, CL and WL drafted the manuscript. All the authors read and approved the final manuscript. All authors contributed to the article and approved the submitted version.

## Funding

This work was supported by grant to WL from the Medical Science and Technology Project of Henan Province (NO. SBGJ2018077). The funder had no role in study design, data collection and analysis, decision to publish, or preparation of the manuscript.

## Conflict of Interest

The authors declare that the research was conducted in the absence of any commercial or financial relationships that could be construed as a potential conflict of interest.

## Publisher’s Note

All claims expressed in this article are solely those of the authors and do not necessarily represent those of their affiliated organizations, or those of the publisher, the editors and the reviewers. Any product that may be evaluated in this article, or claim that may be made by its manufacturer, is not guaranteed or endorsed by the publisher.
