# Supplementary figures and images for: Predictors of Lung Adenocarcinoma With Leptomeningeal Metastases: A 2022 Targeted-Therapy-Assisted molGPA Model
Source: Front Oncol. 2022 Jun 10;12:903851. doi: 10.3389/fonc.2022.903851 (PMC9252592; doi:10.3389/fonc.2022.903851)

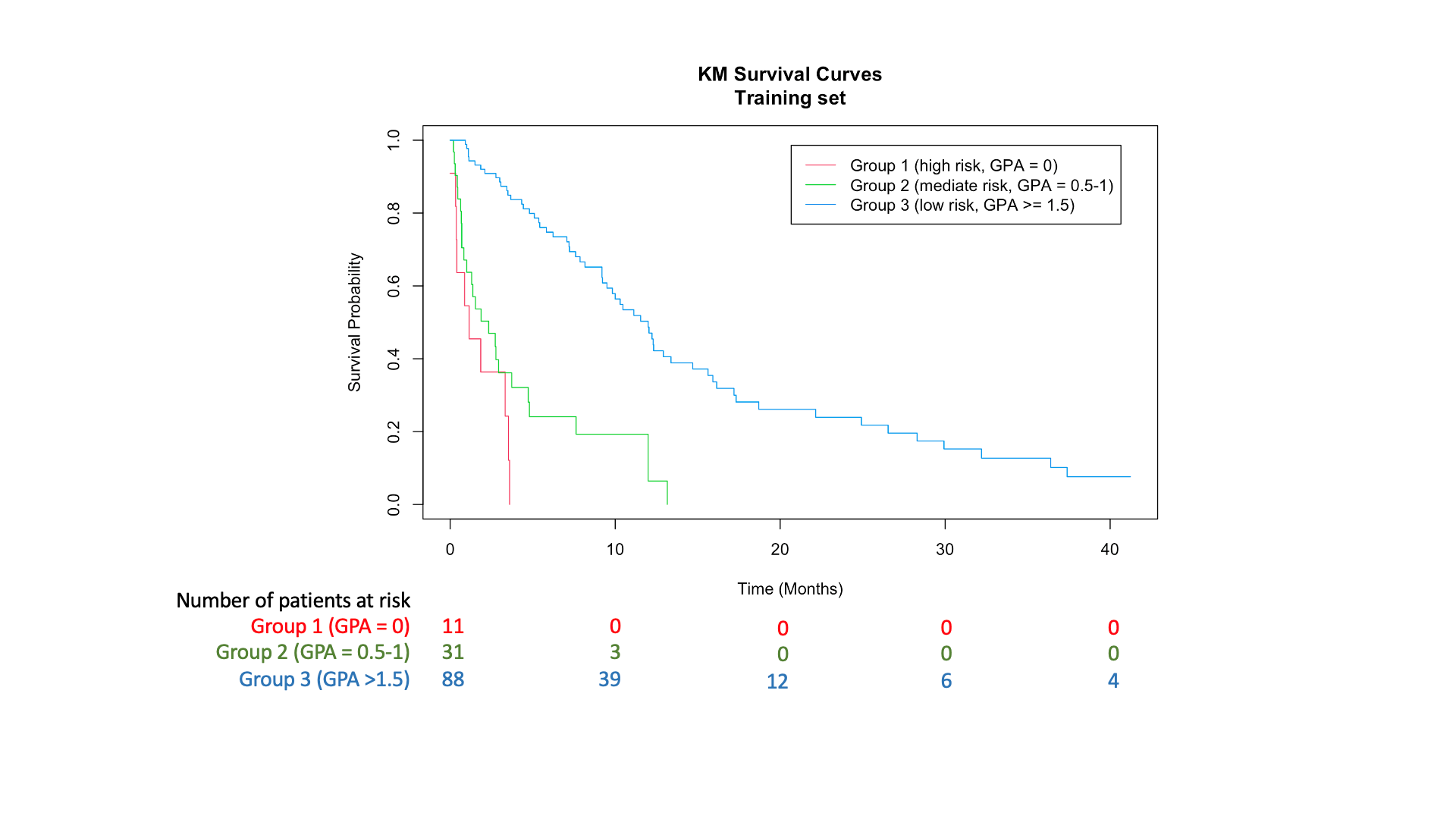

Supplement: Supplementary file 1 [file Image_1.tif]

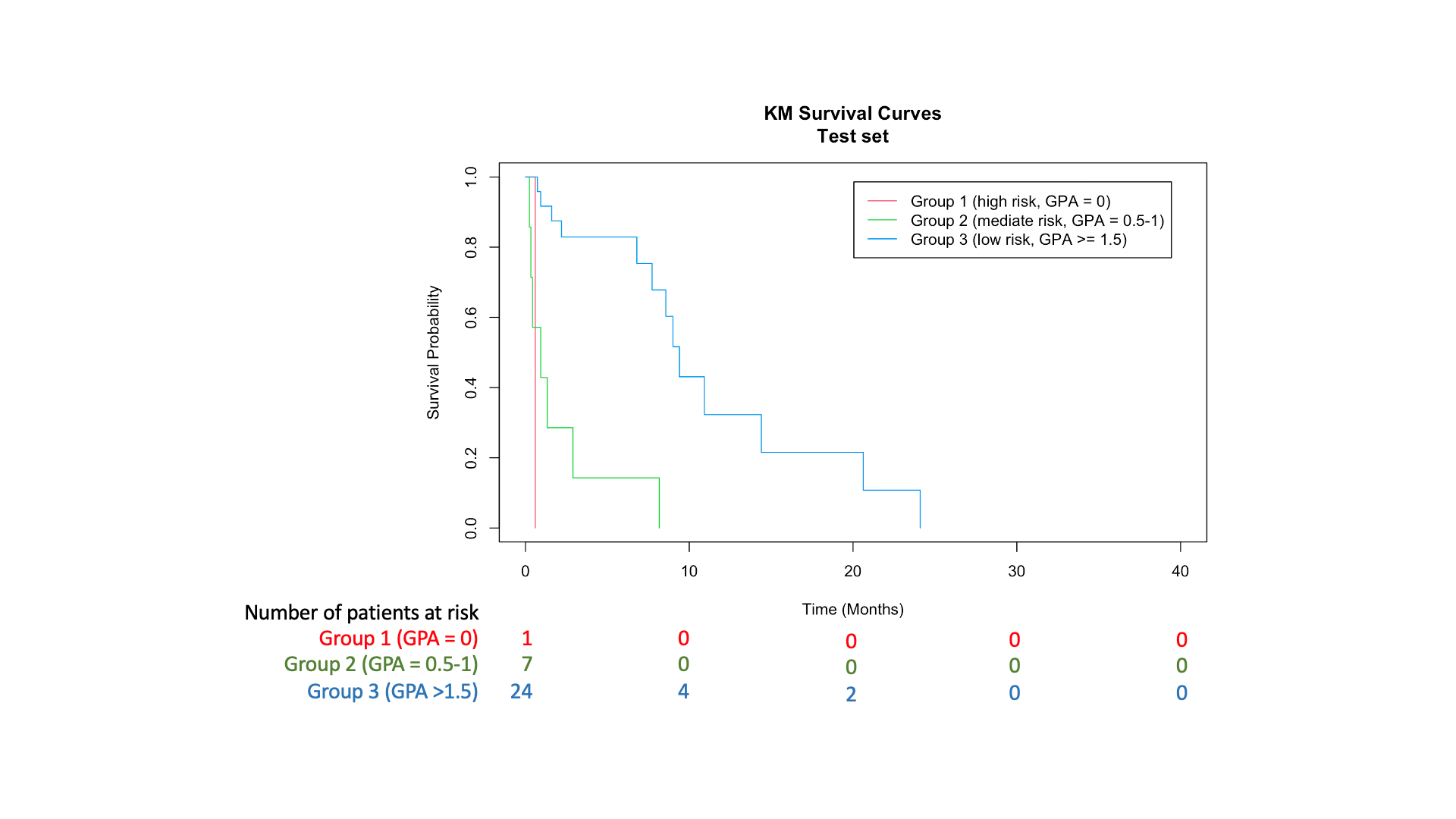

Supplement: Supplementary file 2 [file Image_2.tif]
