# Supplementary material for: Predictors of Lung Adenocarcinoma With Leptomeningeal Metastases: A 2022 Targeted-Therapy-Assisted molGPA Model
Source: Front Oncol. 2022 Jun 10;12:903851. doi: 10.3389/fonc.2022.903851 (PMC9252592; doi:10.3389/fonc.2022.903851)
Supplement: Supplementary file 3 [file Table_1.docx]

**Supplement Table 1. Leptomeningeal Assessment in Neuro-Oncology (LANO) standardized neurological examination**

| Domain | Level of Function Score | | |  | Key Considerations |
| --- | --- | --- | --- | --- | --- |
|  | 0 | 1 | 2 | 3 |  |
| Gait | Normal | Abnormal but walks without  assistance | Abnormal and requires assistance (companion, cane, walker, etc.) | Unable to walk | 1. Walking is ideally assessed by at least  10 steps. |
| Strength | Normal | Movement present but decreased against resistance | Movement present but none against resistance | No movement | 1. Each limb should be tested separately.   2. Recommend assess proximal (above knee or elbow) and distal (below knee or elbow) major muscle groups.  3. Score should reflect worst performing area.  4. Patients with preexisting level 3 function in one major muscle group/limb at baseline can be scored based on assessment of other major muscle groups/limb. |
| Sensation | Normal | Decreased but  aware of sensory  modality | Unaware of sensory modality | --------- | 1. Recommend evaluating major body areas separately (face, limbs, and trunk).  2. Score should reflect worst performing area.  3. Sensory modality includes but is not limited to light touch, pinprick, temperature, and proprioception.  4. Patients with preexisting level 2 function in one major body area at baseline can be scored based on assessment of other major body areas. |
| Vision | Normal | Partial monocular  visual loss | Complete monocular  visual loss | Bilateral visual  loss | 1. Patients who require corrective lenses should be evaluated while wearing corrective lenses.  2. Each eye should be evaluated, and score should reflect worst performing eye. |
| Eye  movements | Normal | Abnormality noted in 1 direction of gaze | Abnormality noted in more than 1 gaze direction, but not all | Unable to move the eye in any gaze direction | 1. Test eye movements for each eye individually.  2. The score will reflect the worst performing eye (ie, the highest score). |
| Facial  strength | Normal | Mild facial weakness (nasolabial fold flattening,  asymmetric smile, decreased forehead contraction, or partial eye closure) | Severe facial weakness (severe nasolabial fold flattening, asymmetric smile with limited or no movement of face, incomplete eye closure, or labial incompetence | Bilateral facial  weakness | 1 Weakness includes nasolabial fold flattening, asymmetric smile, and difficulty elevating eyebrow. |
| Hearing | Normal | Impaired but residual serviceable hearing | Absent unilateral hearing | Bilateral hearing loss | 1. Each ear should be evaluated and score should reflect worst performing ear. |
| Swallowing | Normal | Impaired but not requiring change in diet formulation, not aspirating by bedside testing | Unable to swallow without risk of aspiration by bedside testing | ----------- | 1. Bedside testing comprising a swallow  test with a small glass of water |
| Level of consciousness | Normal | Drowsy (easily arousable &  responsive) | Somnolent (difficult to arouse & poorly responsive) | Coma (unarousable & unresponsive) |  |
| Behavior | Normal | Mild/moderate alteration | Severe alteration | --------- | 1. Alteration includes but is not limited to apathy, disinhibition, and confusion.  2. Consider subclinical seizures for significant alteration. |
| Other | Normal | Occasional or mild | Persistent, moderate  to severe | ------------ | “Other”: Neurological findings not otherwise defined in the current examination, for example ataxia. |

**References:** Marc, Chamberlain, Larry, et al. Leptomeningeal metastases: a RANO proposal for response criteria. Neuro-oncology 2017; 19(4), 484-492.
