# Supplementary material for: Predictors of Lung Adenocarcinoma With Leptomeningeal Metastases: A 2022 Targeted-Therapy-Assisted molGPA Model
Source: Front Oncol. 2022 Jun 10;12:903851. doi: 10.3389/fonc.2022.903851 (PMC9252592; doi:10.3389/fonc.2022.903851)
Supplement: Supplementary file 4 [file Table_2.docx]

**Supplement Table 2. The scoring criteria of the lung-molGPA (2017)**

| Prognostic Factor | Lung-molGPA Scoring Criteria | | |
| --- | --- | --- | --- |
|  | 0 | 0.5 | 1 |
| Age, y | ≥70 | <70 | NA |
| KPS | <70 | 70-80 | 90-100 |
| ECM | Present | - | Absent |
| Brain metastases, No. | ≥4 | <4 | NA |
| Gene status | EGFR neg/unk and ALK neg/unk | NA | EGFR pos or ALK pos |

**Note:** ECM, extracranial metastases; GPA, graded prognostic assessment; KPS, Karnofsky Performance Status; NA, not applicable; neg/unk, negative or unknown; pos, positive; GPA store was categorized into four groups: 0-1.0; 1.5-2.0; 2.5-3.0; and 3.5-4.0.

**Reference:** Sperduto PW, Yang TJ , Beal K, et al. Estimating Survival in Patients With Lung Cancer and Brain Metastases: An Update of the Graded Prognostic Assessment for Lung Cancer Using Molecular Markers (Lung-molGPA). JAMA Oncol 2016; 3(6): 827.
