# Supplementary material for: Predictors of Lung Adenocarcinoma With Leptomeningeal Metastases: A 2022 Targeted-Therapy-Assisted molGPA Model
Source: Front Oncol. 2022 Jun 10;12:903851. doi: 10.3389/fonc.2022.903851 (PMC9252592; doi:10.3389/fonc.2022.903851)
Supplement: Supplementary file 5 [file Table_3.docx]

**Supplement Table 3. The scoring criteria of the molGPA for LM (2019)**

| Prognostic Factor | molGPA Scoring Criteria for LM | | |
| --- | --- | --- | --- |
|  | 0 | 0.5 | 1 |
| KPS | <60 | 60-70 | 80-100 |
| ECM | Present | Absent | - |
| Gene status | EGFR neg/unk and ALK neg/unk | EGFR pos or ALK pos | - |

**Note:** ECM, extracranial metastases; GPA, graded prognostic assessment; KPS, Karnofsky Performance Status; neg/unk, negative or unknown; pos, positive; GPA store was categorized into four groups: 0; 0.5-1.0; 1.5-2.0.

**Reference:** Yin K, Li YS, Zheng MM, et al. A molecular graded prognostic assessment (molGPA) model specific for estimating survival in lung cancer patients with leptomeningeal metastases. Lung Cancer 2019; 131:134-138.
