# Supplementary material for: Predictors of Lung Adenocarcinoma With Leptomeningeal Metastases: A 2022 Targeted-Therapy-Assisted molGPA Model
Source: Front Oncol. 2022 Jun 10;12:903851. doi: 10.3389/fonc.2022.903851 (PMC9252592; doi:10.3389/fonc.2022.903851)
Supplement: Supplementary file 6 [file Table_4.docx]

**Supplement Table 4. Multivariate Cox model with gene mutation status excluding TKI therapy line**

| Variable | HR (95% CI) | P-value |
| --- | --- | --- |
| Controlled primary tumor(1= Yes) | 0.977 (0.615, 1.551) | 0.009 |
| KPS | 0.404 (0.194, 0.842) | 0.046 |
| LANO neurological assessment | 1.106 (1.053, 1.161) | <0.01 |
| Gene mutation status | 0.609(0.590, 0.629) | 0.070 |

**Note:** KPS, Karnofsky performance status; LANO, Leptomeningeal Assessment in Neuro-Oncology; TKI, tyrosine kinase inhibitor.
